# The Mediating Roles of Family Resilience and Social Support in the Relationship Between Illness Severity and Depressive Symptoms Among Primary Caregivers of Children With Epilepsy in China

**DOI:** 10.3389/fneur.2022.831899

**Published:** 2022-02-21

**Authors:** Wenjing Wei, Rongrong Yang, Jie Zhang, Haili Chen, Jinghua Ye, Qiru Su, Jianxiang Liao, Zhitian Xiao

**Affiliations:** ^1^China Medical University, Shenzhen Children's Hospital, Shenzhen, China; ^2^Department of Neurology, Shenzhen Children's Hospital, China Medical University, Shenzhen, China; ^3^Department of Clinical Research, Shenzhen Children's Hospital, China Medical University, Shenzhen, China

**Keywords:** children with epilepsy, caregivers, family, resilience, social support, depression

## Abstract

**Purpose:**

This study was designed to assess the effects of epilepsy severity, family resilience, and social support on depression in primary caregivers of children with epilepsy (CWE), and to test the mediating roles of family resilience and social support in this relationship.

**Method:**

Two hundred fifty-two caregivers of children with epilepsy were recruited from October 2020 to May 2021. The questionnaire contained sociodemographic characteristics, Epilepsy Severity, Chinese-Family Resilience Assessment Scale (C-FRAS), Social Support Rating Scale (SSRS), Beck Depression Inventory (BDI). Structural equation models were used to evaluate whether family resilience and social support as mediators between epilepsy severity and depression.

**Results:**

In this study, the prevalence of depressive symptoms among primary caregivers of CWE in China was 69.84%. Epilepsy severity was positively associated with depression. Family resilience and social support were negatively correlated with depressive symptoms (both *p* < 0.01). Furthermore, the fitness indices of structural models were satisfactory. The direct effect of epilepsy severity on depression was 0.266 (95% CI 0.064–0.458), this pathway explained 62.88% variance of depression. The indirect effect of family resilience and then social support was 0.069 (95% CI 0.025–0.176), indicating that the serial multiple mediation was significant. The serial mediation pathway explained 16.31% variance of depression.

**Conclusions:**

The high incidence of depression among primary carers of CWE deserves more attention. They should be screened routinely, especially those parents of children with severe epilepsy. Family resilience and social support could be protective factors for caregivers' mental adjustment. Therefore, future psychosocial interventions for enhancing family resilience and social support should be implemented, in order to reduce their depression.

## Introduction

Epilepsy is one of the most common chronic neurological disorders in children, which is characterized by recurrent seizures caused by abnormal brain discharge. Approximately 50–70 million people have epilepsy worldwide (1, 2) and the prevalence of epilepsy among children ranges from 3.9 to 5.1‰ in China (3). Epileptic seizures and its treatment not only have a strong negative impact on the children's physical and psychobehavioral development (4, 5), but also exerts detrimental effects on the whole family. Parents often function as children's main caregivers especially for families of CWE in China, they have to deal with these challenges, as well as face high medical costs, stigma from relatives and friends, limited family social interaction, and negative emotional reactions (6, 7). Growing evidence had shown that parents of CWE had a higher risk of depression (8, 9). As Reilly et al. (8) indicated the prevalence of depression in mothers and fathers was 55 and 33%, compared with 27 and 31% correspondingly in the non-epilepsy-related neuro disability group. In China, the risk of depression was higher in parents of CWE compared with healthy children (23.51 vs. 10.84%, *p* < 0.01) (9). Importantly, this psychological distress has been reported to be linked with an increased risk of depression in children, lower health-related quality of CWE, and decreased family function (10–12). Therefore, it is of vital importance to screen the psychological distress among caregivers of CWE and explore its comprehensive influencing factors for providing interventional strategies.

The theory of multifactorial effects of psychological stress and Walsh's family resilience framework highlights that when families face stressors, various factors (i.e., social support, family resources.) could influence the individual's emotional response and family adaption (13, 14). Illness severity, as a major stressor, may be an influential factor for caregivers' depression. Prior researches had found the degree of disease severity was positively correlated with the parental psychological state in families of children with developmental disorders and ASD (15, 16). Furthermore, raising a child with severe epilepsy was highly related to caregivers' distress and depressive symptoms (17). But the latest study showed that the disease severity of CWE cannot predict parental depression in China (18). The relationship between epilepsy severity and depression is contradictory. Therefore, it is necessary to further explore the relationship and potential mediating mechanisms between disease severity of CWE and caregivers' depression.

To confront the effects of negative events on caregivers' depression, family internal resources and external support are essential factors for them to combat depression (19). First, Family resilience, as one of the most critical family resources, refers to the ability to rebound from adversity and become stronger and more resourceful, which comprises shared family faith systems, patterns of organization, and communication or problem-solving processes (14). Chronic illness as a family stressor is not conducive to the development of family resilience (20, 21). And family resilience has been considered as an important source to maintain family members' mental wellbeing. For example, one study indicated that families with high resilience could reduce the risk for parental depression, which explained 14.9% variance of depressive symptoms (22). Meanwhile, available evidence also reveals that family resilience may mediate the relationship between clinical factors in children and family members' mental health. Suzuki et al. (15) found that the relationship between disease severity and depression among mothers of children with developmental disorders can be mediated by family resilience.

Second, social support has been considered as an important external resource in buffering the influence of stress and promoting physical and mental health (23, 24). Social support refers to emotional, informational, or material support provided by professional or non-professional organizations (25). Raising a child with severe seizures can cause caregivers to alienate with extended families and friends, and receive lower social support (26). These situations are negatively associated with their psychological health (27). As previous studies showed that high levels of social support were related to the improvement of psychological wellbeing among mothers of children with autism spectrum disorders (28) and reduction of depressive symptoms in patients with prostate cancer (29). In other words, a powerful support network can assist parents to cope with difficulties and maintain family members' mental wellbeing. As Carlson et al. (30) found social support mediates the relations between perceived epilepsy severity and mothers' anxiety and depression.

**Figure 1:**
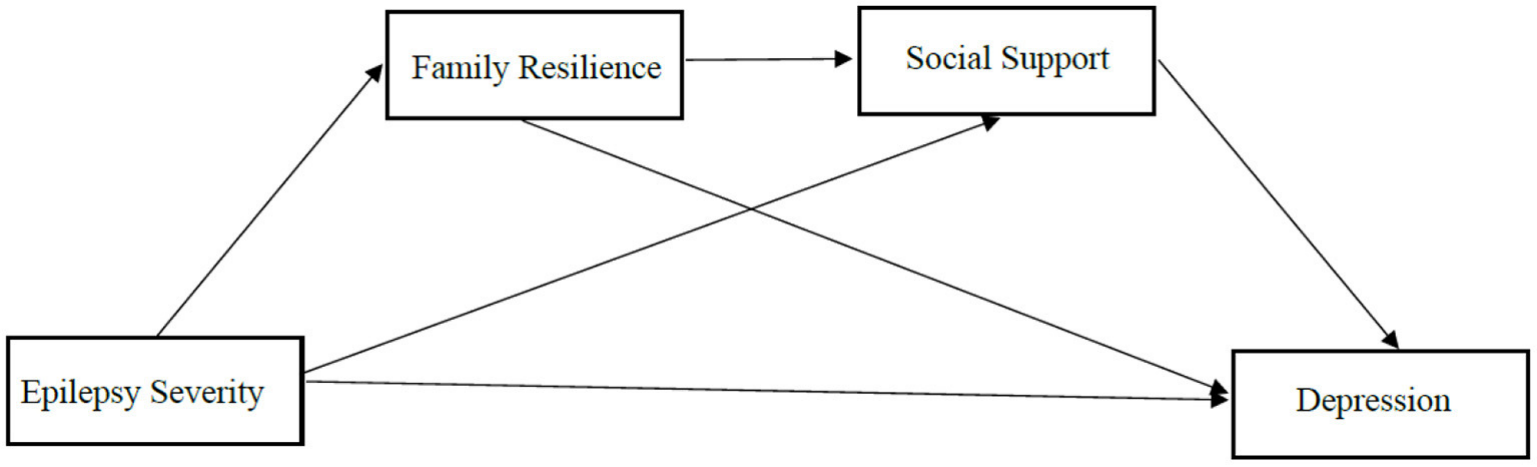
The hypothesized model concerning the relationship between epilepsy severity and depression: family resilience and social support as mediators.

The above studies suggested that family resilience and social support may mediate the relationship between illness severity and caregivers' depression. Nevertheless, to our knowledge, the association of epilepsy severity, family resilience, social support, and depression have not been investigated among parents of CWE in China. Moreover, whether family resilience and social support mediate the association between epilepsy severity and depression remains unexplored. Accordingly, this study was aimed to evaluate the depressive symptoms among primary caregivers of CWE as well as explore the potential effects of family resilience and social support in the relationship between epilepsy severity and caregivers' depression. The theoretical framework was developed based on existing studies, see Figure 1. We used data collected from primary caregivers of CWE to test the three hypotheses.

*Hypothesis* 1: The depressive symptoms among primary caregivers of CWE was common, and higher levels of epilepsy severity increased the risk for parental depression.
*Hypothesis* 2: When families faced adversities, higher levels of family resilience and social support played a vital role in decreasing the rate of depression among primary caregivers of CWE.
*Hypothesis* 3: The relationship between epilepsy severity and depression was mediated by family resilience and social support among primary caregivers of CWE.

## Materials and Methods

### Participants

Two hundred fifty-two caregivers of CWE in the neurology ward and neurology outpatient were recruited from a tertiary hospital in Guangdong Province. The inclusion criteria for participants were: (1) mothers or fathers of CWE and primary caregiver (Assuming the primary responsibility for caregiving the child, living with and taking care of the child for at least 72 h per week, or at least 12 h per day); (2) having a child aged 0–14 years, and diagnosed with epilepsy by neurologists according to the International League Against Epilepsy (ILAE) criteria (31); (3) aged ≥ 18 years. The exclusion criteria included: (1) the child diagnosed with other complications and (2) principal caregivers were diagnosed with severe medical conditions or cognitive impairment or mental illness. (3) Moreover, families were also excluded if the family experienced traumatic events such as serious natural disasters, accidents, and sudden death of relatives in the past half-year. All parents participated in the study voluntarily and signed the informed consent.

### Procedure

Ethical approval was provided by the Medical Ethics Committee of Shenzhen Children's Hospital (No. 2020067), following the Declaration of Helsinki. Data were collected using convenience sampling methods during October 2020 and May 2021. After obtaining written informed consent, all participants were asked to complete questionnaires independently in the neurology wards or neurology outpatient waiting rooms. The questionnaire included four parts: sociodemographic characteristics of children and primary caregivers, family resilience, social support, and depression. The entire survey took about 20–30 min to complete. A total of 280 primary caregivers of CWE were recruited to complete the questionnaire, eighteen caregivers refused to participate, ten participants who filled out questionnaires incompletely were excluded. Thus, 252 (96.18%) participants completed the entire and valid questionnaire.

### Instrument

#### Sociodemographic Characteristics

The self-designed questionnaire was used to collect basic demographic characteristics of CWE and their primary caregivers. The data included patients' gender, age, duration of epilepsy. The information of primary caregivers included their relationship with the child, age, residence, occupation, income per month, education, religion, medical payment. These were mainly collected by medical records and self-report of parents.

#### Epilepsy Severity

Epilepsy severity was used to measure childhood epilepsy. The total scores of illness severity are 1–9, determined by seizure types (1-3), frequency of seizures (0–3), and the number of anti-seizure medications (ASMs) used (0–3). We assigned a score to the seizure types, 3 for generalized tonic-clonic seizures, 2 for partial seizures, and 1 for the absence of seizures. If the children have weekly or daily seizures, the score is 3, 2 for monthly seizures, 1 for once or twice per year, and 0 for no seizures during the previous year. A score of 0 is assigned when the children have no medication, 1 for single ASMs, 2 for two ASMs, and 3 for three or more ASMs. The three scores are summed, 1–5 is considered low epilepsy severity, and ≥6 is considered high epilepsy severity (32, 33). In this study, the Cronbach's α was 0.605, which was acceptable. These data were collected from the medical records.

#### Chinese-Family Resilience Assessment Scale

The Chinese-Family Resilience Assessment Scale (C-FRAS) was used to evaluate the resilience levels of families (34). The 44-item scale includes four dimensions: family communication and problem solving (FCPS), utilizing social and economic resources (USR), maintaining a positive outlook (MPO), and the ability to make meaning of adversity (AMMA). It uses a Likert four-point scale from strongly disagree to strongly agree (1–4), with a total score of 44–176. Higher scores indicate higher degrees of family resilience. The Cronbach's α of C-FRAS was 0.960, and the four subscales Cronbach's α range from 0.70 to 0.97 (34). In this study, the Cronbach's α was 0.958, 0.946, 0.888, 0.884, and 0.807 for C-FRAS, FCPS, USR, MPO, and AMMA.

#### Social Support Rating Scale

Social Support Rating Scale (SSRS) (35) was used to measure the degree of support received from friends, relatives, and healthcare providers. The 10-item self-rated scale contains three subscales: objective support, subjective support, and support utilization. Among them, the scores for items 5, 6, and 7 are based on the number of choices, and other items are scored on four-point scale. The higher scores indicate higher levels of social support. The Cronbach's α was 0.707 for SSRS in the present study.

#### Beck Depression Inventory

The Beck Depression Inventory (BDI) was used to detect the severity of depressive symptoms within the past week (36). BDI has 21 items, each item is scored from 0 to 3 based on self-assessment severity, which total scores ranging from 0 to 63. Higher scores reflect the increasing severity of depressive symptoms. Scores of 5–13 were considered mild depression, scores of 14–20 showed moderate depressive symptoms, and scores equal or above 21 indicated severe depressive symptoms. In this study, the Cronbach's α of this scale was 0.849.

### Statistical Analysis

EpiData 3.1 was used to input the data and IBM SPSS Statistics (version 25.0, IBM Corp, Armonk, NY, USA) was used to perform statistical analysis. Two-sided *p*-value < 0.05 was statistically significant. The demographic characteristics and four main variables (epilepsy severity, family resilience, social support, and depression) were analyzed descriptively. Continuous data were described as means ± standard deviation (SD) or median (interquartile range Q1–Q3) according to whether the data follows a normal distribution. Categorical data are described using frequencies and percentages. Pearson correlations were used to explore the relations among these variables. Principal caregiver, monthly family income, occupation, medical expenses payment were included as control variables.

Structural Equation Modeling (SEM) was used to examine the mediating effect of family resilience and social support. The maximum likelihood (ML) procedure was used given the variables were normally distributed, which was inferred by skewness (±3) and kurtosis (±8). For latent variables (i.e., epilepsy severity, family resilience, social support), we used the domain-representative approach to get items parcels. And random assignment approach to get items parcels for depression in Excel (37).Chi-square/degrees of freedom (χ^2^/df), Comparative Fit Index (CFI), Tucker-Lewis index (TLI) and Incremental Fit Index (IFI), Root Mean Square Error of Approximation (RMSEA) were used to evaluate the fit of the model. Ninety-five percentage bootstrap confidence interval (CI) was used to estimate the significance of the indirect effect. The mediation effect was significant if the 95% CI did not contain 0. SEM was running in AMOS 26.0 (IBM Corp., Armonk, NY, USA).

## Results

### Demographic Characteristics and Four Variables

Among 252 parents of children with epilepsy, 201 (79.80%) were mothers, accounting for a high proportion, and 51 (20.20%) were fathers, the average age was (35.41 ± 5.06) years, with a range of 23 to 48 years. Children with epilepsy had a mean age of (5.83 ± 3.87) years, ranging from 0 to 14 years, with the median disease duration being 24 months (IQR 10–48). The prevalence of depression was 69.84%, including mild, moderate, and severe depression. As shown in Table 1.

**Table 1:** Descriptive statistics for sociodemographic characteristics and depression (*N* = 252).

**Variable**	**Response**	***N* (%)**
Child gender	Male	144 (57.1)
	Female	108 (42.9)
Age of children	≤ 3	88 (34.9)
(years old)	3–6	56 (22.2)
	7–14	108 (42.9)
Principal caregiver	Mother	201 (79.8)
	Father	51 (20.2)
Residence	Countryside	48 (19.0)
	Suburban	27 (10.7)
	City	177 (70.2)
Occupation	Employed	152 (60.3)
	Unemployed	100 (39.7)
Religion	Yes	26 (10.3)
	No	226 (89.7)
Monthly family income	<5,000	27 (10.7)
(Yuan)	5,000–10,000	70 (27.8)
	10,000–15,000	53 (21.0)
	>15,000	102 (40.5)
Education	High school or below	92 (36.5)
	Undergraduate	150 (59.5)
	Graduate or above	10 (4.0)
Medical expenses payment	Urban basic medical insurance	145 (57.5)
	New rural cooperative medical insurance	57 (22.6)
	Self-paying and others	50(19.8)
Depression	Mild	92(36.5)
	Moderate	45(17.8)
	Severe	39(15.5)

In Table 2, the average score of depression was (10.96 ± 9.25), and epilepsy severity was (5.55 ± 2.07), 141(55.95%) of children were low epilepsy severity, 111 (44.05%) of children were high epilepsy severity. The average score of family resilience was (134.96 ± 16.65), family communication and problem solving was rated highest, while utilizing social and economic resources received the lowest score. The average score of social support was (38.69 ± 6.04), with the domain of objective support received the highest scores, followed by subjective support, and utilization of support was the lowest.

**Table 2:** Description statistics and correlations among the study variables (*N* = 252).

	**Number of items**	**Mean ±SD**	**1**	**2**	**3**	**4**	**5**	**6**	**7**	**8**	**9**	**10**	**11**
1. Epilepsy severity	9	5.55 ± 2.07	1										
2. C-FRAS	44	134.96 ± 16.65	−0.247^**^	1									
3. FCPS	27	85.70 ± 11.12	−0.227^**^	0.967^**^	1								
4. USR	8	21.73 ± 3.50	−0.217^**^	0.713^**^	0.561^**^	1							
5. MPO	6	18.23 ± 3.03	−0.210^**^	0.831^**^	0.732^**^	0.519^**^	1						
6. AMMA	3	9.30 ± 1.25	−0.148^*^	0.714^**^	0.634^**^	0.442^**^	0.676^**^	1					
7. Social support	10	38.69 ± 6.04	−0.221^**^	0.477^**^	0.440^**^	0.418^**^	0.384^**^	0.336^**^	1				
8. OS	4	21.73 ± 3.94	−0.078	0.254^**^	0.258^**^	0.156^*^	0.198^**^	0.167^**^	0.658^**^	1			
9. SS	3	10.18 ± 2.50	−0.252^**^	0.468^**^	0.420^**^	0.438^**^	0.394^**^	0.314^**^	0.852^**^	0.254^**^	1		
10. US	3	6.78 ± 1.68	−0.085	0.237^**^	0.211^**^	0.240^**^	0.161^*^	0.220^**^	0.614^**^	0.281^**^	0.335^**^	1	
11. Depression	21	10.96 ± 9.25	0.374^**^	−0.385^**^	−0.373^**^	−0.290^**^	−0.296^**^	−0.284^**^	−0.404^**^	−0.199^**^	−0.377^**^	−0.268^**^	1

** *p < 0.01*;

* *p < 0.05*.

*C-FRAS, Chinese-Family Resilience Assessment Scale; FCPS, Family Communication and Problem Solving; USR, Utilizing Social and economic Resources; MPO, Maintaining a Positive Outlook; AMMA, Ability to Make Meaning of Adversity; OS, Objective Support; SS, Subjective Support; US, Utilization of Support; SD, standard deviation*.

### Correlations Between Epilepsy Severity, Family Resilience, Social Support, and Depression

The correlation analysis results were summarized in Table 2, which showed significant correlations among these variables. Epilepsy severity was negatively correlated with family resilience (*r* = −0.247, *p* < 0.01) and social support (*r* = −0.221, *p* < 0.01). According to the effect size criteria of Cohen (23), these effects were weak. Epilepsy severity was positively related to depression (*r* = 0.374, *p* < 0.01). Family resilience and social support were negatively correlated with depression (*r* = −0.385, *r* = −0.404, respectively, *p* < 0.01), with a moderate effect size. These bivariate correlations suggest that the following mediation analysis can be performed.

### Validation of Structural Model

We used SEM to test the model, with epilepsy severity as an independent variable, family resilience and social support as the mediating variables, and caregivers' depression as the dependent variable. SEM results demonstrated that the structural model had a good fit to the data (38), with χ^2^/df = 1.801, CFI = 0.933, IFI = 0.934, TLI = 0.919, RMSEA = 0.056.

### Mediating Effects of Family Resilience and Social Support in the Relationship Between Epilepsy Severity and Depression

**Figure 2:**
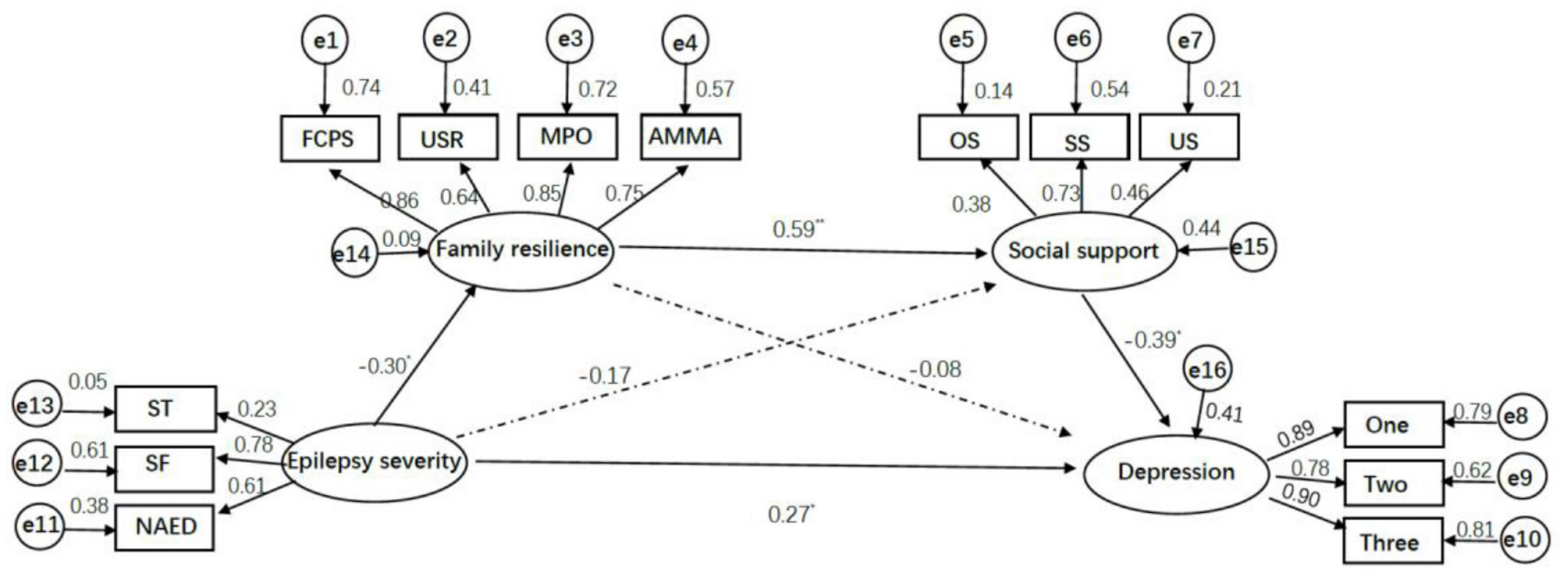
Structural equation model of epilepsy severity, family resilience, social support, and depression. *p < 0.01, ** *p* < 0.001. FCPS, USR, MPO, AMMA, four parcels of family resilience; FCPS, Family Communication, and Problem Solving; USR, Utilizing Social and economic Resources; MPO, Maintaining a Positive Outlook; AMMA, Ability to Make Meaning of Adversity; ST, SF, NASM, three parcels of epilepsy severity; ST, Seizure Type; SF, Seizures Frequency; NASM, number of anti-seizure medications; OS, SS, US, three parcels of social support; OS, objective support; SS, subjective support; US, utilization of support; One, Two, Three, three parcels of depression using random assignment approach.

As presented in Figure 2, the standardized coefficient of epilepsy severity on family resilience was β = −0.298, *p* < 0.01, and family resilience on depression was β = −0.078, *p* > 0.05, and the indirect effect of this pathway was 0.023. The 95% CI for indirect effect from epilepsy severity to depression *via* family resilience was −0.042 to 0.094, the 95% CI included zero, indicating the indirect effect of this pathway was not statistically significant.

The standardized coefficient of epilepsy severity on social support was β = −0.166, *p* > 0.05, and social support on depression was β = −0.390, *p* < 0.01. The indirect effect of this pathway was 0.065, 95% CI (−0.006, 0.220), which indicated the indirect effect of social support was also not statistically significant.

The standardized coefficient of family resilience on social support was β = 0.593, *p* < 0.001, the serial mediation effect from epilepsy severity to depression through family resilience and then social support was 0.069, 95% CI (0.025, 0.176). We concluded that there was a significant serial mediation effect. In addition, the direct effect of epilepsy severity on depression was 0.266, 95% CI (0.064, 0.458), *p* < 0.05, indicating the existence of a direct effect.

The total indirect effect of these three pathways was 0.157, 95% CI (0.073, 0.319), which explained the 37.12% variance of depression. Of which, the serial mediation pathway explained 16.31% variance of depression, and the direct effect pathway explained 62.88% variance of depression. As shown in Table 3.

**Table 3:** The model path diagram, total indirect effect, total effect analysis of the four concepts.

				**Bootstrap**		
				**95%CI**		
**Path**	**Effect size**	**S.E**.	***P***	**Lower**	**Upper**	**Effect proportion (%)**
Epilepsy severity -> Family resilience->Depression	0.023	0.034	0.379	−0.042	0.094	5.44%
Epilepsy severity->Social support->Depression	0.065	0.055	0.070	−0.006	0.220	15.37%
Epilepsy severity->Family resilience->Social support ->Depression	0.069	0.035	0.001	0.025	0.176	16.31%
Epilepsy severity->Depression	0.266	0.100	0.010	0.064	0.458	62.88%
Total indirect effect	0.157	0.061	0.001	0.073	0.319	37.12%
Total effect	0.423	0.084	<0.001	0.252	0.585	

## Discussion

In this study, we developed a multiple-mediation model between epilepsy severity and caregivers' depression to investigate the protective roles of family resilience and social support against negative effects on caregivers' psychological adjustment. Our study confirmed that depressive symptoms was common among parents of CWE in China. And epilepsy severity was positively correlated with depressive symptoms (supporting hypothesis 1). Meanwhile, it also corroborated that family resilience and social support could reduce the risk for depression (supporting hypothesis 2). Importantly, there was a serial mediation pathway between severity and depression through family resilience and then social support (partly supporting hypothesis 3).

In the present study, the prevalence of depressive symptoms was 69.84%. A recent cross-sectional research conducted among 308 caregivers of children with epilepsy found that the proportion of depression accounts for 65.60% (18), which was consistent with our reported incidence of depression. In other studies, the prevalence of depressive symptoms ranged from 23.5 to 55% (8, 9). A possible explanation for this discrepancy is the difference in instruments. In addition, the higher incidence of depressive symptoms in the current study may attribute to the mean age of CWE in this study is (5.83 ± 3.87) years and the median disease duration is 24 months, indicating earlier onset in children with epilepsy. As shown in previous studies, early-onset epilepsy was often associated with intractable seizures, developmental delay, and a high risk for epileptic encephalopathy (39), which inevitably had a detrimental effect on parental mental health (8). Meanwhile, the high incidence of depression could be related to the fact that limiting the study to parents of CWE rather than other relatives. Prior studies indicated that parents are more likely to experience psychological burden and parenting stress, which will increase the risk for depression (6, 40). Our study further supports that all parents of CWE should be screened for depression (8).

In terms of the relationship between epilepsy severity and caregivers' depression, Phillips et al. (41) demonstrated that caregivers of children who gained seizure freedom had fewer depressive symptoms compared with caregivers of children with consistent seizures. This could be attributed to that parent of children with severe epilepsy experience more physical, psychological, and economic burdens (26). However, a Danish study assessing the incidence of psychopathology in parents of children with high-severity epilepsy reported that seizure-related factors were not related to caregivers' mental distress (17). In the present study, we demonstrated that disease severity was positively correlated with caregivers' depression, that is caregivers of children with low-severity epilepsy have fewer depressive disorders. A possible explanation for this difference is that the evaluation of key aspects of epilepsy severity varied among studies. Conducting qualitative research may be helpful to elucidate the nature of the relations between epilepsy severity and parental depression.

Inconsistent with our expectations, family resilience and social support were not independently mediated the relationship between illness severity and depression. While the serial mediation of family resilience and then social support was found among primary caregivers of CWE in the present study. These results further validated the theory of multifactorial effects of psychological stress and Walsh's family resilience framework. As Jiang et al. (13) indicated psychological stress response is actually a system of multiple factors interacting with each other, which ultimately affects the individuals' mental health. This may partly explain why family resilience and social support cannot independently mediate the relationship between illness severity and depression.

In addition, our finding differs from prior studies, which found family resilience and social support as independent mediators among mothers of children with developmental disorders in Japan (15), and mothers of children with epilepsy in the USA (30). The possible reason for this difference is that children with epilepsy affect caregivers' mental adaptation beyond the effects of family resilience and social support alone. For example, due to social misconceptions and negative attitudes, epilepsy is regarded as a kind of mental illness in China, the families often experience severe stigma, especially in rural areas (42). This is considered as the greatest handicap for people with epilepsy rather than the disability caused by recurrent seizures, causing families tremendous psychological burden (43–45). Furthermore, there are still no respite care services for CWE in China, caring for CWE is regarded as parents' priority. They have to give up social activities to take care of their children, and the effects on their mental health outweigh the severity of epilepsy (46). Finally, families of children with epilepsy have difficulty developing supportive and sharing parent-child relationships (47), and they are more prone to experience marital disharmony than caregivers of children without epilepsy (48). These crises could weaken the ability of families to recover from the difficulties. External support is essential for maintaining the mental health of family members.

Noteworthily, the serial mediation pathway between epilepsy severity and depression through family resilience and then social support was found among primary caregivers of CWE in the present study. In other words, family with children of low-severity epilepsy can maintain higher levels of resilience than others, which promote the mobilization of social resources. Therefore, the primary caregivers would experience lower depression. This is possibly due to that families have a positive outlook toward crises, a flexible family organization model, open and clear communication, which enables them better take advantage of social support (49, 50). Meanwhile, family resilience and social support could positively predict the individuals' psychological resilience, which further contributes to maintaining individuals' mental health in the face of stressful events (51). The serial mediation analysis provides another comprehensive evidence that epilepsy severity impacts parents' psychological adjustment through family resilience and social support. Family resilience and social support are modifiable factors that can be assessed at the initial medical visit. By identifying the needs of the primary caregivers and providing proper support for the whole family to improve the parental mental wellbeing.

Based on these findings, health professions can provide interventions in the effort to minimize parental depressive symptoms by identifying multiple factors. For example, Puka et al. found that online mindfulness-based intervention programs can significantly improve the CWE's and parents' mental wellbeing. This program includes mindful awareness, social-emotional learning skills, and positive psychology (52). In addition, interventions aimed to enhance family resilience include family narrative co-construction, systemic family therapy (foster shared family beliefs, problem-solving skills, coping strategies, fostering hope, and communication) (49, 53). Health professionals can also assist families to explore available social resources to further establish family-community-society support networks.

There exists three limitations. First, our study enrolled participants from a single center in China, the representativeness of samples is limited. In other words, the external validity of our results may be limited by the difference in the characteristics of caregivers from different regions. Multi-center, larger samples studies should be conducted in the future. Second, due to the cross-sectional design of the study, we could not infer the causality relations and dynamic changes over time among variables. Cohort studies can be conducted in the future to explore the mediate effect of these variables at different stages. Third, we measured family resilience only through one caregiver of the children with epilepsy, which could not fully reflect family functions. Therefore, it is recommended that assess family resilience from the perspective of children with epilepsy and other family members in the future.

## Conclusion

To our knowledge, this is the first study to explore the complex interactions between epilepsy severity, social support, family resilience, and mental condition among parents of CWE in China. We found that the incidence of depression among primary caregivers of CWE reached 69.84%, and epilepsy severity was positively correlated with caregivers' depression. Importantly, our study confirmed the serial mediation effects of family resilience and social support in the relationship between epilepsy severity and depression. This finding may be helpful in determining treatment strategies, where families living with children of high-severity epilepsy are more likely to benefit from interventions designed to strengthen family resilience and social support. This may reduce the negative impact of epilepsy severity on caregivers' mental health.

## Data Availability Statement

The original contributions presented in the study are included in the article/supplementary material, further inquiries can be directed to the corresponding author.

## Ethics Statement

The studies involving human participants were reviewed and approved by Medical Ethics Committee of Shenzhen Children's Hospital. The patients/participants provided their written informed consent to participate in this study.

## Author Contributions

WW and ZX designed the study. RY, JZ, HC, and JY were involved in data collection. WW and QS analyzed the data. WW wrote the original draft. JL and ZX provided a critical review of the original draft. All authors read and approved the content of the manuscript.

## Funding

This study was supported by the Sanming Project of Medicine in Shenzhen (SZSM201812005), Shenzhen Key Medical Discipline Construction Fund (No. SZXK033), and the Shenzhen Fund for Guangdong Provincial Highlevel Clinical Key Specialties (No. SZGSP012).

## Conflict of Interest

The authors declare that the research was conducted in the absence of any commercial or financial relationships that could be construed as a potential conflict of interest.

## Publisher's Note

All claims expressed in this article are solely those of the authors and do not necessarily represent those of their affiliated organizations, or those of the publisher, the editors and the reviewers. Any product that may be evaluated in this article, or claim that may be made by its manufacturer, is not guaranteed or endorsed by the publisher.
